# Incorporation of Fluorescence Ceramide-Based HPLC Assay for Rapidly and Efficiently Assessing Glucosylceramide Synthase *In Vivo*

**DOI:** 10.1038/s41598-017-03320-9

**Published:** 2017-06-07

**Authors:** Sachin K. Khiste, Salman B. Hosain, Yixuan Dong, Mohammad B. Uddin, Kartik R. Roy, Ronald A. Hill, Zhijun Liu, Yong-Yu Liu

**Affiliations:** 1 Department of Basic Pharmaceutical Sciences, University of Louisiana at Monroe, Monroe, LA 71209 USA; 2 School of Renewable Natural Resources, Louisiana State University Agriculture Center, Baton Rouge, LA 70803 USA

## Abstract

Glucosylceramide synthase (GCS) is a rate-limiting enzyme catalyzing ceramide glycosylation, thereby regulating cellular ceramide levels and the synthesis of glycosphingolipids (GSLs) in cellular membranes. Alterations of GCS not only affect membrane integrity, but also closely correlate with stem cell pluripotency, cancer drug resistance, GSL storage disorders and other diseases. Enzyme activities measured conventionally with currently available *ex-vivo* methods do not enable reliable assessment of the roles played by GCS *in vivo*. We report herein a substrate-incorporation method enabling rapid and efficient assessment of GCS *in-vivo* activity. Upon nanoparticle-based delivery, fluorescent NBD C6-ceramide was efficiently converted to NBD C6-glucosylceramide in live cells or in mouse tissues, whereupon an HPLC assay enabled detection and quantification of NBD C6-glucosylceramide in the low-femtomolar range. The enzyme kinetics of GCS in live cells and mouse liver were well-described by the Michaelis-Menten model. GCS activities were significantly higher in drug-resistant cancer cells and in tumors overexpressing GCS, but reduced after silencing GCS expression or inhibiting this enzyme. Our studies indicate that this rapid and efficient method provides a valuable means for accurately assessing the roles played by GCS in normal *vs*. pathological states, including ones involving cancer drug resistance.

## Introduction

Glucosylceramide synthase (GCS; EC2.41.80) catalyzes ceramide glycosylation, transferring glucose residues from UDP-glucose to ceramide (Cer) and thereby producing glucosylceramide (GlcCer)^1, 2^. Cer glycosylation by GCS is the first and rate-limiting step for the synthesis of glycosphingolipids (GSLs), and GlcCer serves as a foundational substrate for further elaboration via glycosylations, thus providing the core structure for more than 400 GSLs in vertebrates^3–5^. Highly localized to GSL-enriched microdomains (GEMs) of cellular membranes, GSLs play crucial roles in modulating membrane integrity and functions, including cell-cell recognition and communication^3, 6, 7^.

In addition to serving as building blocks of membranes, GSLs modulate cell adhesion and cellular signaling. Inhibition of GCS, thereby decreasing GlcCer accumulation, is a treatment option for Gaucher’s disease^8^ and type II diabetes^9, 10^. GSLs are involved in cell proliferation, differentiation, immuno-response and oncogenic transformation^11–13^. Cer, as a lipid second messenger, mediates many cell-stress responses in embryo development and in processes combatting tumorigenesis^14–17^. Moreover, Cer-induced apoptosis is highly correlated to the efficacy of anticancer regimens, such as those involving anthracyclines, taxanes, *Vinca* alkaloids, TNF-α or radiation therapy^18–22^. Recent studies concordantly indicate that enhanced expression of GCS is a cause of cancer drug resistance^23–28^. Inhibition of Cer glycosylation through targeting of GCS thus emerges as a promising therapeutic approach for improving outcomes of cancer treatments^19, 27, 29, 30^.

Quantitative assessment of GCS activity is essential for evaluating the roles Cer glycosylation plays in cell functions, as well as in the therapeutic efficacies of relevant disease treatments. After Basu’s work^1^, several additional methods have been reported^2, 31–33^. Besides those assays relying on the radioactivity of UDP-[^3^H]glucose^31, 34, 35^ for detection, *N*-[6-[(7-nitro-2,1,3-benzoxadiazol-4-yl) amino]hexanoyl]-d-*erythro*-sphingosine (NBD C6-Cer) has been used as an acceptor for Cer glycosylation catalyzed by GCS^2, 32, 36^. The HPLC method based on NBD C6-Cer has proven to be a highly sensitive and reproducible assay for assessing GCS activity *in vitro* with optimal conditions^2, 32^. Convergently, previous studies have shown that NBD C6-Cer can be used as an exogenously supplied substrate for characterizing cellular Cer glycosylation and assessing GCS activities with thin-layer chromatography (TLC) and spectrometry^28, 37, 38^. With nanoparticle based delivery of NBD C6-Cer, we developed a rapid, efficient, and fully quantitative substrate incorporation HPLC analysis for assessing GCS *in-vivo* activity in live cells and in living mice.

## Results

### NBD C6-Cer incorporation-based HPLC analysis of ceramide glycosylation

**Figure 1:**
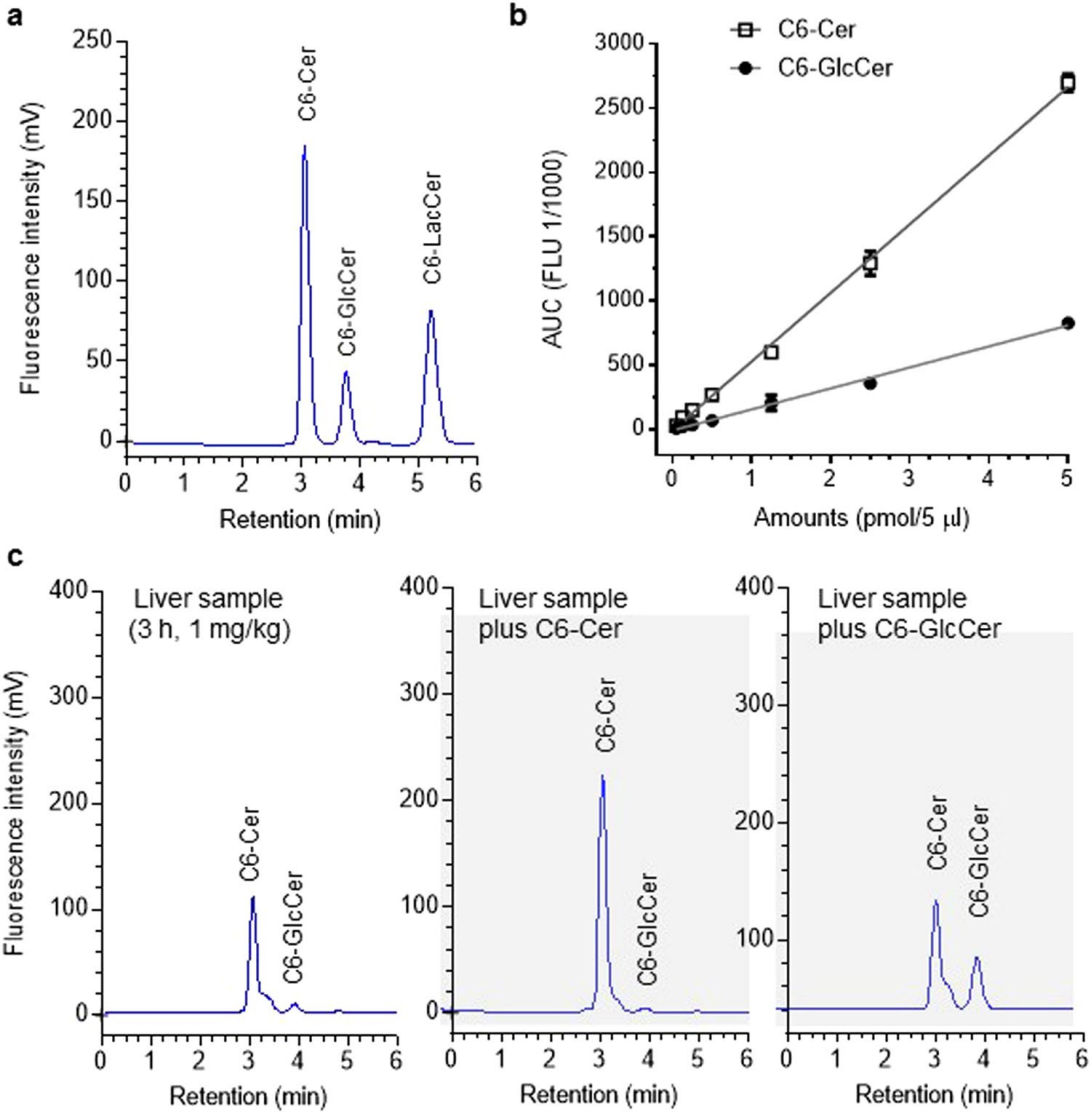
HPLC analysis of NBD C6-Cer and C6-GlcCer. (**a**) HPLC chromatogram of sphingolipids. A mixed standard of NBD C6-Cer, C6-GlcCer and C6-LacCer (1:1:1, 0.5 pmol each) was separated and quantitated by HPLC with fluorescence detection. The retention times for C6-Cer, C6-GlcCer and C6-LacCer were 3.1, 3.9, and 5.2 min, respectively. (**b**) Quantitation of C6-Cer and C6-GlcCer. Increasing amounts of mixtures of NBD C6-Cer and C6-GlcCer (1:1) were resolved and analyzed by HPLC. Each data point represents mean ± SD of three independent experiments. The correlation coefficients for NBD C6-Cer and NBD C6-GlcCer were 0.99 and 0.99, respectively. (**c**) Characterization of NBD C6-Cer and C6-GlcCer in mouse liver. Lipids extracted from livers of mice after administration of NBD C6-Cer-RUB (1 mg/kg, i.p.; 180 min) were spiked with NBD C6-Cer (0.25 pmol) or C6-GlcCer (0.25 pmol) and analyzed by HPLC.

A cell-permeable NBD C6-Cer BSA complex was employed for delivery of NBD C6-Cer to cells^37^. GCS converts NBD C6-Cer to NBD C6-glucosylceramide (C6-GlcCer), accompanying glycosylation of endogenous ceramide in the Golgi apparatus. To quantitatively characterize Cer glycosylation in cells, NBD C6-Cer and NBD C6-GlcCer levels were assessed by HPLC using calibration curves prepared from authentic NBD C6-Cer and NBD C6-GlcCer. As shown in Fig. 1a, mixtures of NBD C6-Cer/C6-GlcCer/C6-LacCer (1:1:1, 0.5 pmol each) were effectively separated on a normal-phase column (5 µm ZORBAX Rx-SIL 4.6 × 250 mm) using a binary linear gradient formed from solvent system A (chloroform/methanol/*ortho*-phosphoric acid, 80:20:0.1, v/v/v) and solvent system B (chloroform/methanol/H_2_O/*ortho*-phosphoric acid, (60:34:6:0.1, v/v/v/v) at a flow rate of 1.0 ml/min (Fig. 1a). NBD C6-Cer, NBD C6-GlcCer and NBD C6-LacCer (lactosylceramide) eluted with retention times of 3.1, 3.9, and 5.2 min, respectively. Additionally, galactosylceramide (GalCer) could also be identified, although its peak eluted very closely to that of GlcCer, as shown in brain samples spiked with NBD C6-GlcCer or NBD C6-GalCer (Fig. S1).

To quantitate NBD C6-Cer and NBD C6-GlcCer, the samples containing both were serially diluted and analyzed. As shown in Fig. 1b, fluorescence intensity areas-under-the-curves (AUCs) of NBD C6-Cer and NBD C6-GlcCer peaks correlated well with amounts loaded over a wide concentration range (50 to 5,000 fmol in 5.0 µl) (Fig. 1b), with correlation coefficients for NBD C6-Cer and NBD C6-GlcCer of 0.99 and 0.99 (*r* ^2^ of linear regression, *p* < 0.001), respectively. The minimum amounts of NBD C6-Cer and NBD C6-GlcCer detected by this HPLC analysis were 50 fmol. We further confirmed the identities of these analytes in tissue samples by spiking them with NBD C6-Cer and NBD C6-GlcCer standards. As shown in Fig. 1c, added NBD C6-Cer (0.25 pmol) or NBD C6-GlcCer (0.25 pmol) precisely merged with the C6-Cer and C6-GlcCer peaks in liver samples obtained from mice 3 h after administration of NBD C6-Cer-rubusoside (NBD Cer-RUB, 1 mg/kg, intraperitoneal injection).

**Figure 2:**
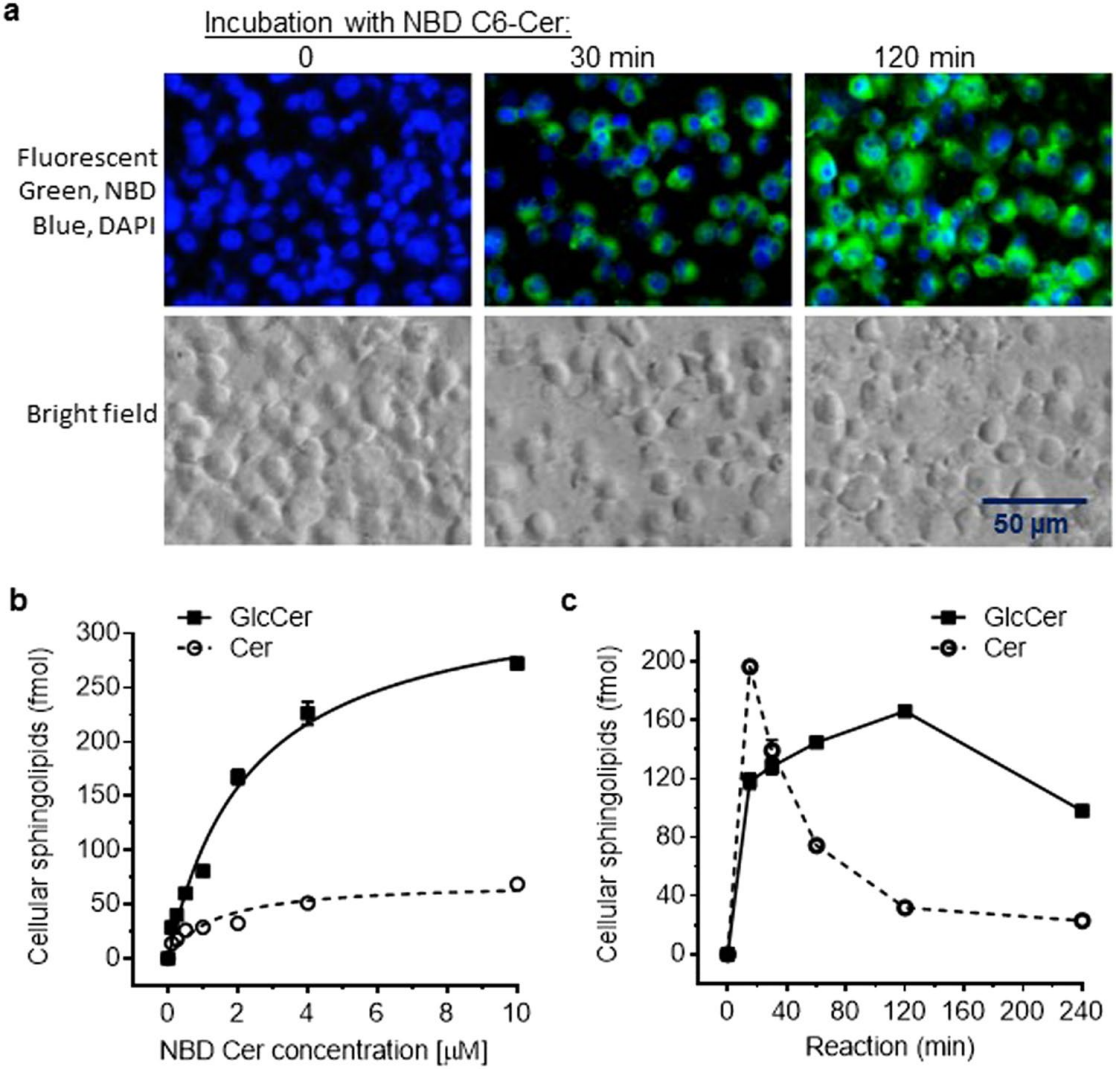
Determination of NBD C6-Cer glycosylation in cells. NCI/ADR-RES cells cultured for 24 hr were switched to 1% BSA RPMI-1640 medium containing NBD C6-Cer for enzymatic reactions. (**a**) Fluorescent NBD-sphingolipids in cells after incubations with 2 µM NBD C6-Cer. Green, NBD-sphingolipids; Blue, DAPI-nuclei. Images (200× magnification) were captured using an EVOS FL cell imaging system. (**b**) Dose-response for Cer glycosylation by GCS. Cells were incubated with NBD C6-Cer for 2 h. The correlation coefficient for cellular C6-Cer (dashed line) to NBD C6-Cer in medium is 0.91, and for cellular C6-GlcCer (solid line) to NBD C6-Cer in medium is 0.99. (**c**) Time course of cellular Cer glycosylation. Cells were incubated with 2.0 µM of NBD C6-Cer for indicated periods. The correlation coefficient for cellular C6-GlcCer (solid line) to NBD C6-Cer in medium is 0.99. Results represent the mean ± SD of three independent enzyme reactions.

Intracellular Cer glycosylation by GCS was verified using human NCI/ADR-RES cells that express high levels of GCS^25, 37, 39^. For maximum cellular uptake, cells were incubated with NBD C6-Cer complexed to bovine serum albumin (BSA) in RPMI-1640 medium containing 1% BSA. After 120-min incubations with 2 µM NBD C6-Cer, cellular fluorescence attributable to NBD C6-sphingolipids was significantly increased (Fig. 2a). With increasing concentrations of NBD C6-Cer (0.1–10 µM) in the incubation medium, we found that cellular concentrations of NBD C6-Cer and NBD C6-GlcCer, substrate and product of ceramide glycosylation, respectively, increased correlatively (Fig. 2b; *r* ^2^, 0.91, 0.99) and in accord with Michaelis-Menten behavior. Under these experimental conditions, the K_m_ value calculated for human GCS was 2.34 µM, with a V_max_ of 343 fmol/µg-2 h. These data indicate that this NBD C6-Cer incorporation-based HPLC method is capable of sensitive quantitative assessment of cellular ceramide glycosylation.

The dynamics of cellular NBD C6-Cer concentrations mainly depend on cellular uptake and on conversion of NBD C6-Cer to glycosphingolipids (GSLs). The ability to accurately assess GCS rests primarily on sufficiency of time for the initial production of robustly detectable levels of NBD C6-GlcCer upon incubation. To ascertain an optimal time for routine GCS activity analysis, NCI/ADR-RES cells were incubated with NBD C6-Cer (2.0 µM) for different periods of time (Fig. 2c). Intracellular NBD C6-Cer levels sharply increased, reaching a peak at 15 min, indicating rapid cellular uptake. NBD C6-Cer levels subsequently decreased, with accordant appearance of NBD C6-GlcCer. After an initiation phase, NBD C6-GlcCer levels continued to increase, reaching saturation at 2 h. Thereafter, NBD C6-GlcCer levels decreased (see 4 h time-point) as other GSLs produced from GlcCer increased (Fig. S2). This finding likely indicates induced downstream enzymes (including lactosylceramide synthase) catalyzing the conversion of NBD C6-GlcCer into other glycosphingolipids at higher rates. This observation is also consistent with previous studies showing that GCS is the first rate-limiting enzyme in aggregate GSL synthesis^5, 27^. Based on this kinetic characterization, we selected 2-h incubation of cells with 2 µM NBD C6-Cer in 1% BSA RPMI-1640 medium for further GCS assays.

### Validation of HPLC-based characterization of Cer glycosylation in cell lines expressing different levels of GCS

**Figure 3:**
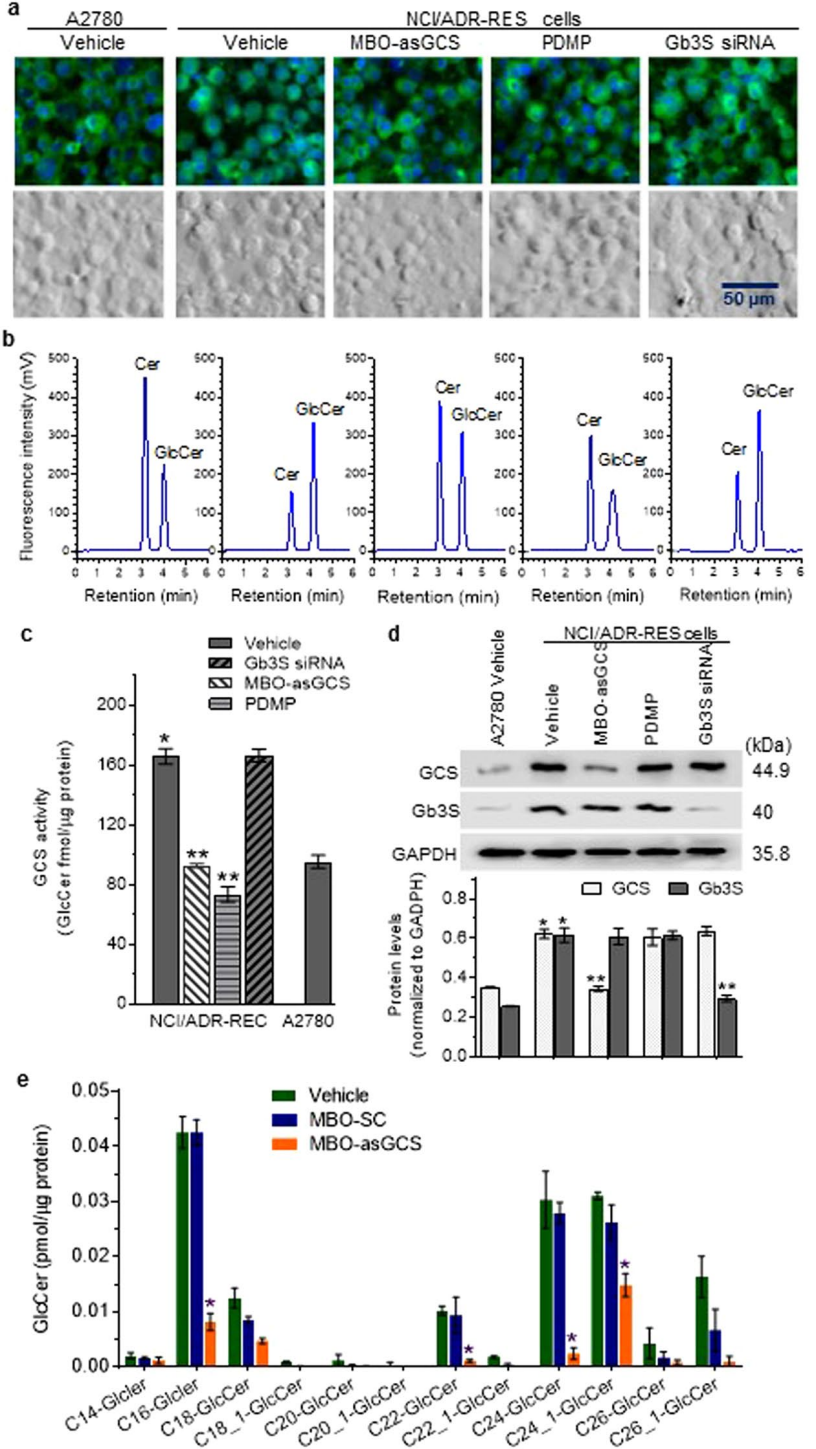
Glycosylation of NBD C6-Cer in cell lines expressing different levels of GCS. Glycosylation of NBD C6-Cer (2 µM, 2 h) was carried out with drug-sensitive A2780 ovarian cancer cells and drug-resistant NCI/ADR-RES cells after indicated treatments. (**a**) Fluorescent sphingolipids in cells after incubation with NBD C6-Cer. Green, NBD-sphingolipids; Blue, DAPI-nuclei. **(b)** Chromatograms of cellular sphingolipids. Cer and GlcCer were identified by their retention times, comparing with NBD C6-Cer and C6-GlcCer standards. (**c**) Intracellular GCS activities. *p < 0.001, compared to A2780 cells; **p < 0.001 compared to NCI/ADR-RES vehicle. Results are the mean ± SD of three independent experiments. (**d**) Western blotting of GCS protein. Equal amounts of detergent-soluble proteins (50 µg protein/lane) were resolved using 4–20% gradient SDS-PAGE, and then immunoblotted with antibodies for GCS or Gb3S and GAPDH. Protein levels of GCS and Gb3S are represented as ratios of GCS/GAPDH optical densities averaged from three blots. *p < 0.001, compared to A2780 cells; **p < 0.001 compared to vehicle. (**e**) Intracellular GlcCer speciation by ESI/MS/MS. Lipids from NCI/ADR-RES cells treated with MBO-asGCS (100 nM, 48 h) or MBO-SC (scrambled control) were analyzed, and the levels of GlcCer in samples expressed as fmol/µg protein, as normalized against total cellular protein. *p > 0.05 compared to MBO-SC or vehicle control.

Cellular conversion of Cer to GlcCer is governed mainly by the status of GCS. To validate the developed methodology for GCS activity assessment, we used drug-sensitive A2780 and drug-resistant NCI/ADR-RES ovarian cancer cell lines, as previous studies indicated that higher levels of GCS constitute the basis of drug resistance in cells of the latter line^26, 28, 39^. NCI/ADR-RES cells were treated with MBO-asGCS (100 nM) to suppress GCS expression^39^, or with PDMP (10 µM) to inhibit GCS enzyme activity^40^. In further verifying this method for GCS assessment, NCI/ADR-RES cells also were treated with siRNA-Gb3S to silence the expression of globotriaosylceramide synthase (Gb3S) or alpha-1,4-galactosyltransferase^28^, another glycosyltransferase involved in GSL synthesis. After 2-h incubation with NBD C6-Cer, NBD C6-sphingolipids substantially accumulated in A2780 and NCI/ADR-RES cells (Fig. 3a). HPLC analysis identified NBD C6-GlcCer in all samples (Fig. 3b). The GCS activity in NCI/ADR-RES cells was approximately 1.7-fold higher than in A2780 cells (166 *vs*. 95 fmol/µg-2 h, *p* < 0.001), which is consistent with the observed GCS protein levels in these two cell lines (Fig. 3c,d). Suppression of GCS expression with MBO-asGCS in NCI/ADR-RES cells significantly decreased GCS activity, by 1.8-fold (93 *vs*. 166 fmol/µg-2 h, *p* < 0.001), in accord with the observed decrease of GCS protein levels (Fig. 3c,d). The GCS activity was also significantly reduced, by more than 2.3-fold (73 *vs*. 166 fmol/µg-2 h, *p* < 0.01), in NCI/ADR-RES cells treated with 10 µM PDMP, without alteration of GCS protein expression (Fig. 3c,d). Gb3S siRNA transfection, which can silence Gb3S expression^28^ (Fig. 3d), did not have any significant effect on either GCS expression (Fig. 3d) or GCS enzyme activity in NCI/ADR-RES, as compared to the vehicle control (Fig. 3c).

GCS activity in NCI/ADR-RES cells after MBO-asGCS treatment was further assessed using LC/MS analysis of endogenous GSLs. MBO-asGCS significantly reduced GCS activity, to approximately 20% of the control, based on the levels of total GlcCer species. Concentrations of several species of GlcCer, including C16-GlcCer, C22-GlcCer, C24-GlcCer, and C24_1-GlcCer, were reduced significantly, by more than 5-, 7-, 8-, and 2-fold, respectively, compared to vehicle treatment (Fig. 3e). Collectively, these results indicate that the NBD C6-Cer incorporation based HPLC method can directly assess GCS activity, with specificity, in cells that are associated with altered cellular responses to anticancer drugs.

### Determination of *in vivo* GCS activities in tissues

**Figure 4:**
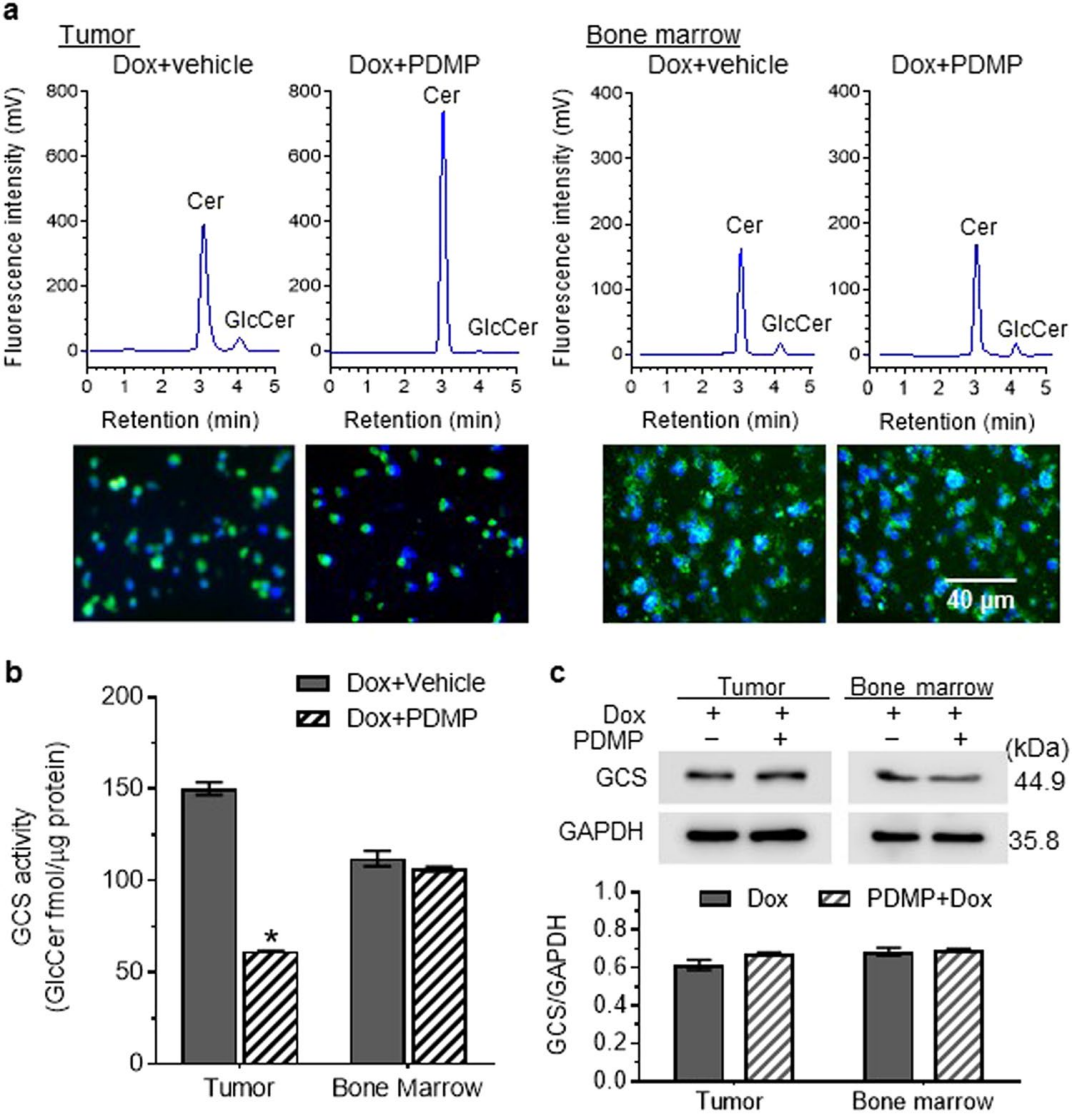
Cer glycosylation by GCS in tumors and tissues. Mice bearing SW48/TP53 tumors were treated with doxorubicin (Dox) alone or combined with PDMP (4 mg/kg, every 3 days for 30 days; 5 cases/group). Cell suspensions of tumors and bone marrow (5 cases/group) were freshly prepared and incubated with NBD C6-Cer (2 µM, 2 h). (**a**) HPLC chromatograms and intracellular NBD sphingolipids. Cer and GlcCer were identified by retention times *vs*. NBD C6-Cer and C6-GlcCer standards. Green, NBD sphingolipids; Blue, DAPI-nuclei. Images (200× magnification) were captured using an EVOS FL cell imaging system. (**b**) GCS activities in tumor and bone marrow. *p < 0.001, as compared to Dox alone. (**c**) Western blotting of GCS in tumor and bone marrow. Equal amounts of detergent-soluble proteins (50 µg protein/lane) extracted were resolved using 4–20% gradient SDS-PAGE and then immunoblotted with antibodies for GCS or GAPDH. GCS protein levels are represented as ratios of GCS/GAPDH optical densities averaged from three blots.

We applied this method to assess GCS activity in mice-borne tumors generated by inoculation with SW48/TP53 cells that had become resistant to doxorubicin (Dox)^41^. Mice were treated with PDMP (4 mg/kg, *i.p*., every 3 days) and Dox (200 µg/kg, *i.p*., every 6 days) for 30 days. Cell suspensions of tumors and bone marrow prepared freshly were incubated with NBD C6-Cer (2.0 µM, 2 h) in 1% BSA RPMI-1640 medium for assessment of Cer glycosylation. As shown in Fig. 4a, NBD sphingolipids accumulated in these cell suspensions; HPLC analysis detected NBD C6-Cer and NBD C6-GlcCer, and no significant amounts of any other GSLs, in all tumor and bone marrow samples. It was found that GCS activity decreased by more than 2.5-fold (62 *vs*. 150 fmol/µg-2 h, *p* < 0.001) in tumors of mice treated with PDMP combined with Dox, as compared to treatment with Dox alone (Fig. 4b), with no discernible change in GCS protein expression (Fig. 4c). In contrast, GCS activities were *not* significantly changed in bone marrow cells of mice treated with Dox combined with PDMP, as compared treatment with Dox alone (Fig. 4b,c).

**Figure 5:**
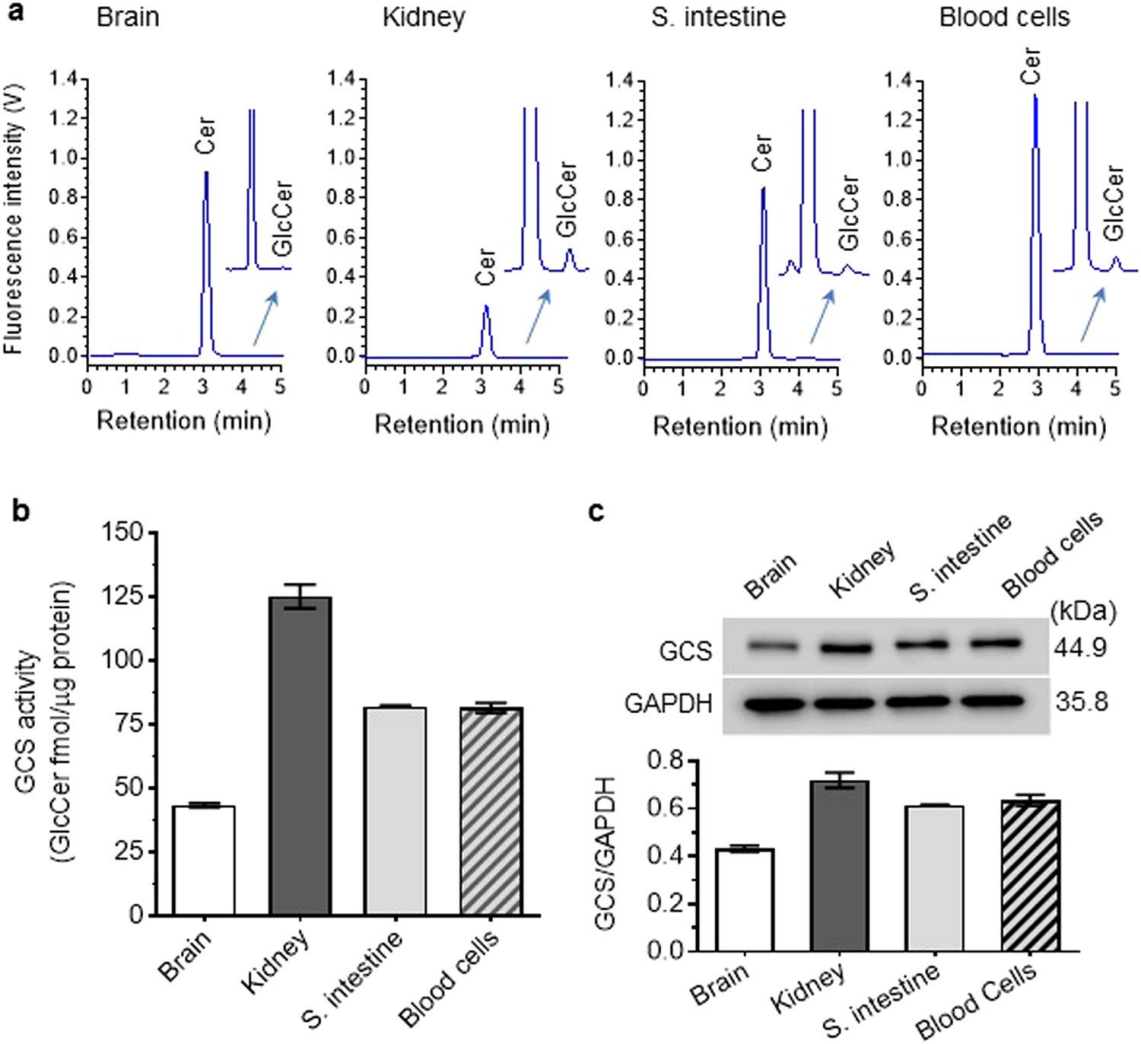
Cer glycosylation by GCS in mouse tissues. (**a**) HPLC chromatograms. Cell suspensions of tissues freshly prepared (from 3 mice) were incubated with NBD C6-Cer (2 µM, 2 h), and the lipids analyzed by HPLC. Arrows indicate enlarged parts of the original chromatograms. (**b**) GCS activities in tissues. (**c**) Western blotting of GCS protein. S. intestine, small intestine. Equal amounts of proteins (50 µg protein/lane) were resolved, and then immunoblotted with antibodies for GCS or GAPDH. GCS protein levels are represented as ratios of GCS/GAPDH optical densities averaged from three blots.

We characterized GCS activities in cell suspensions prepared from other tissues of mice. NBD C6-Cer and NBD C6-GlcCer were seen in brain, kidney, small intestine and blood cells, but GlcCer production was substantially less than in tumors or bone marrow of tumor-bearing mice (Figs 4a and 5a). The GCS activity in brain is approximately 3-fold lower (43 *vs*. 125 fmol/µg-2 h) than kidney, and 2-fold (43 *vs*. 82 fmol/µg-2 h) lower than small intestine and blood cells (Fig. 5b). These results are in accord with Western blot characterization, which indicates that GCS protein levels in whole brain are less than in the other tissues examined (Fig. 5c).

### Direct and efficient assessment of GCS in mice

**Figure 6:**
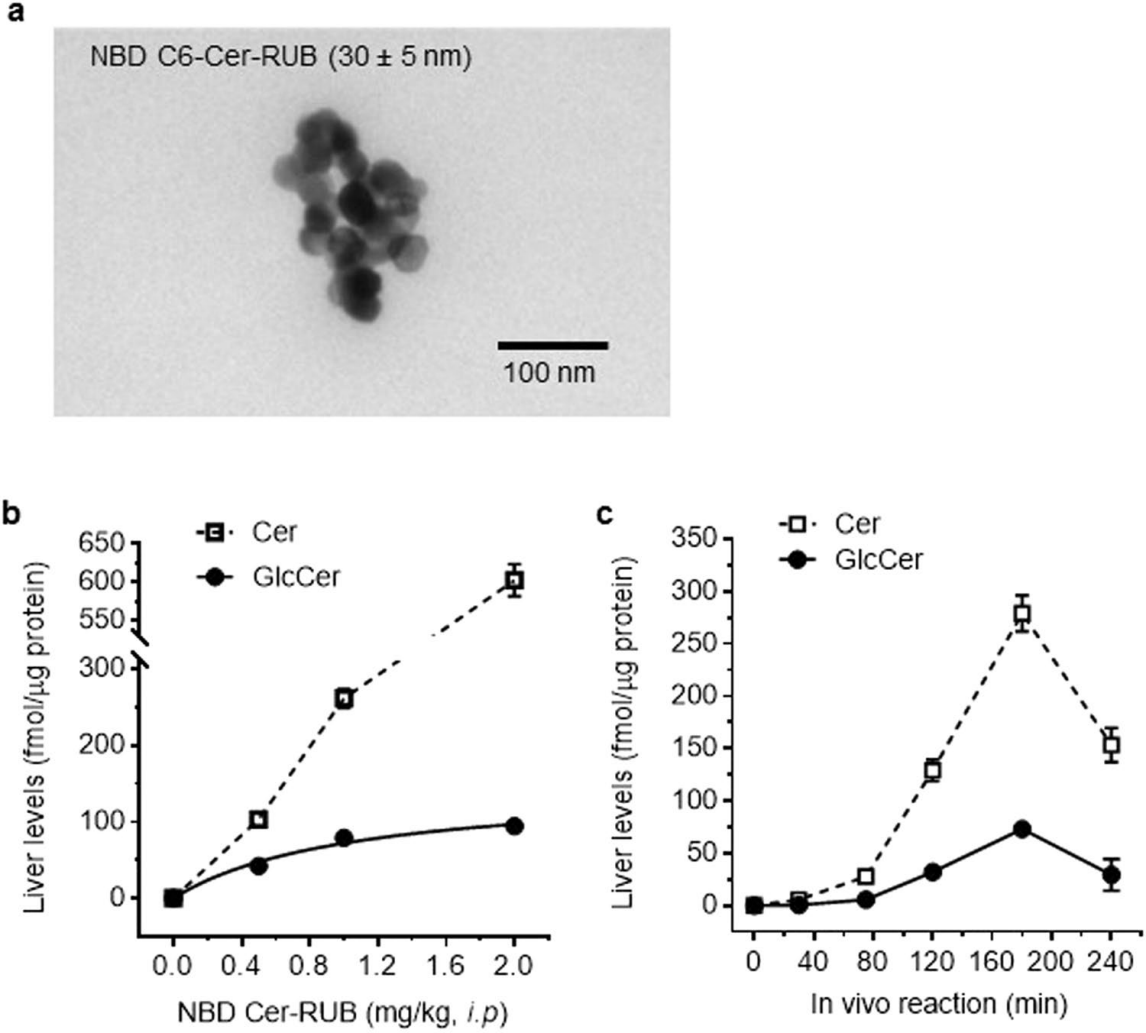
NBD C6-Cer-RUB nanomicelles and ceramide glycosylation in mouse liver. (**a**) Transmission electron microscopy (TEM) of NBD C6-Cer-RUB nanomicelles. A representative TEM image revealed the formation of Cer-RUB nanomicelles with an average size of 30 ± 5 nm. Cer-RUB nanomicelles were dissolved in RPMI-1640 medium and intraperitoneally injected into mice, and 3 h after administration, liver was excised and lipids immediately extracted for HPLC analysis. (**b**) Dose-response for intra-organ Cer glycosylation in mice. NBD C6-Cer-RUB nanomicelles (0.5–2.0 mg/kg) were intraperitoneally injected into mice. The correlation coefficient for C6-Cer (dashed line) to NBD C6-Cer-RUB injected is 0.99, and for C6-GlcCer (solid line) is 0.98. (**c**) Time course of intra-organ Cer glycosylation. Liver lipids were extracted at indicated times following NBD C6-Cer-RUB administration (1 mg/kg). Results represent the mean ± SD of three independent experiments.

To explore the possibility of sensitively and directly characterizing Cer glycosylation in live animals, we generated NBD Cer-RUB nanomicelles and directly characterized NBD Cer uptake and intra-organ glycosylation in mice. NBD C6-Cer-RUB is water-soluble (>0.2%), with an average particle size of 30 nm as measured by transmission electron microscopy (TEM) (Fig. 6a). After 180-min administrations of NBD C6-Cer-RUB (0.5–2 mg/kg, i.p.) in mice, we extracted lipids and found upon HPLC analysis that liver NBD C6-Cer and NBD C6-GlcCer were increased correlatively to the amounts of NBD C6-Cer-RUB injected (Fig. 6b,c; *r* ^2^ = 0.99, 0.98, respectively). The levels of GlcCer produced increased dose-proportionately at lower doses of NBD Cer-RUB injected, but then approached saturation, in accord with Michaelis-Menten behavior (Fig. 6c). The K_m_ value calculated for liver GCS under these conditions is 1.05 mg/kg (equal to 1.82 µM), with a V_max_ of 148 fmol/µg-3 h. Intra-organ (liver) NBD C6-Cer began to increase strongly 80 min after NBD C6-Cer-RUB injections (1 mg/kg, i.p.) (Fig. 6c), reaching a peak at 180 min, a 155-min delay as compared to the time-course seen in cultured cells (Fig. 2c). Thereafter, Cer levels decreased due to GlcCer production. These kinetic characteristics indicate that nanomicelles efficiently delivered NBD C6-Cer in liver, and furthermore, that sampling 3 h after i.p. administration (1 mg/kg, i.p.) constitutes an optimal time-point for measuring glycosylation as an index of cellular GCS activity in tissues.

**Figure 7:**
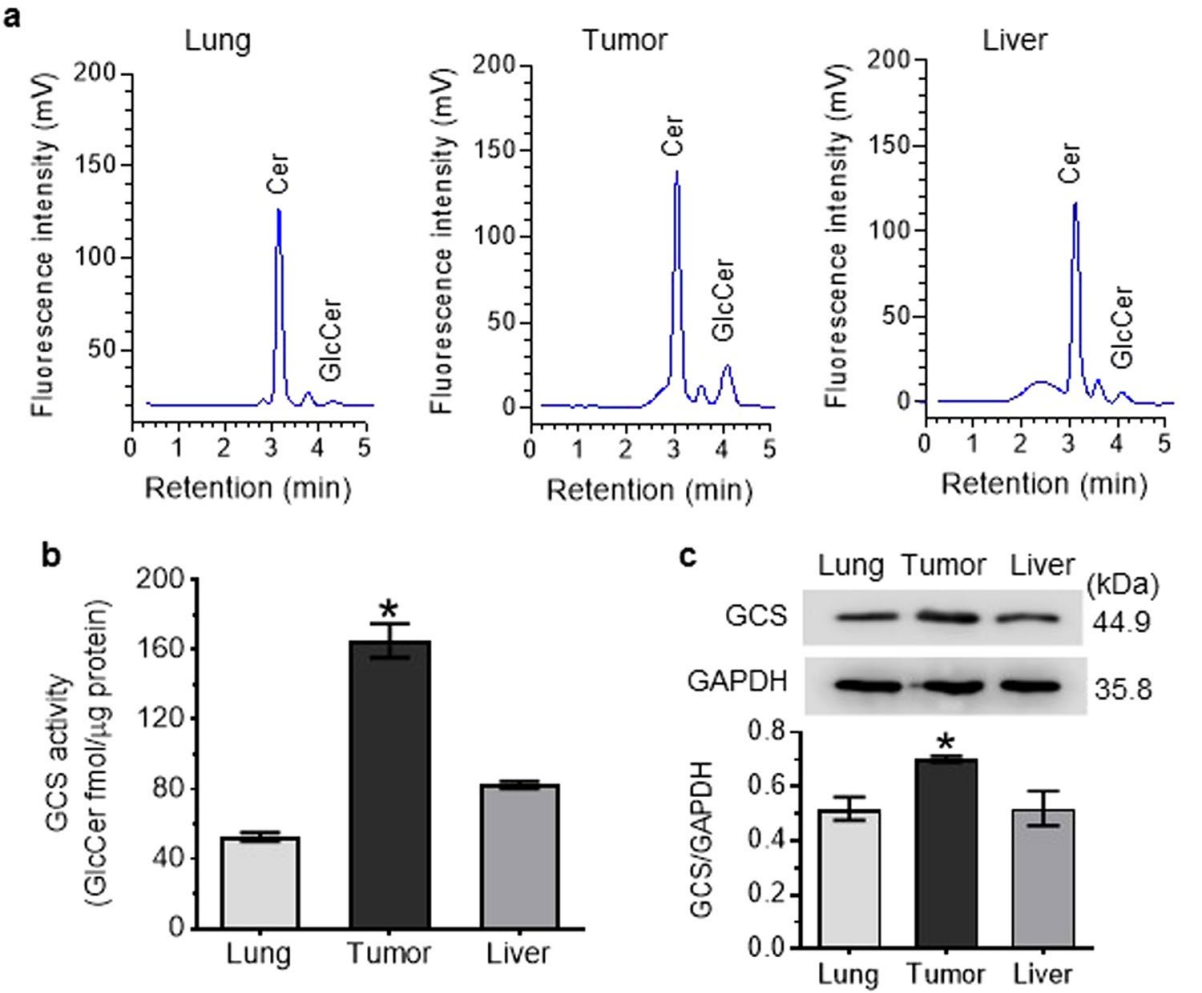
Intra-organ Cer glycosylation by GCS in mice. (**a**) HPLC chromatograms. NBD C6-Cer-RUB nanomicelles (1 mg/kg, i.p.; 4 cases/group) were administered to mice bearing an OVCAR-3 tumor xenograft; 3 h after injection, tissues were rapidly resected for lipid extraction and HPLC analysis. Approximately equal-fluorescence-unit samples of extracted sphingolipids were analyzed by HPLC. (**b**) Intra-organ GCS activities. *p < 0.001 compared to lung or liver. (**c**) Western blotting of tissue GCS. Equal amounts of proteins (50 µg protein/lane) extracted were resolved and then immunoblotted with antibodies for GCS or GAPDH. GCS protein levels are represented as ratios of GCS/GAPDH optical densities averaged from four blots. *p < 0.05 compared to lung or liver.

We also assessed intra-organ Cer glycosylation in mice carrying tumors generated from OVCAR-3 cancer cells. Tumors and other tissues were resected 3 h after administration of NBD C6-Cer-RUB (1 mg/kg, i.p), and the extracted lipids were analyzed by HPLC. NBD Cer-RUB nanomicelles (1 mg/kg, *i.p*.) effectively delivered NBD C6-Cer into a number of tissues, including lung, liver, and xenograft-generated tumors, wherein NBD C6-Cer was glycosylated to NBD C6-GlcCer and other GSLs (Fig. 7a). GCS activities in OVCAR-3 tumors were found to be 3-fold higher than in lung (165 *vs*. 53 fmol/µg), and 2-fold higher than in liver (165 *vs*. 82 fmol/µg) (Fig. 7b). Western blotting concordantly found GCS protein levels in tumors to be higher than in lung and liver (Fig. 7c).

## Discussion

In the present work, we developed a sensitive method with which to quantitate Cer glycosylation catalyzed by GCS *in vivo*, thereby providing a direct and efficient assessment of *in vivo* GCS activity for evaluating the roles played by GCS in cell processes. Assessing enzyme activities (not just protein expression levels) in cells, or furthermore in tissues, is essential for identifying and verifying the actual actions of enzymes in physiological functioning and as relates to their disease-associated roles; however, such assessment in native (*in-vivo*) environments is considerably more difficult than assays conventionally performed under laboratory-controlled conditions. Firstly, identification of a substrate not present endogenously, and which is reliably detectable along with its conversion product with sufficient analytical selectivity and sensitivity, is essential. NBD C6-Cer, which is cell-permeable, has been used to characterize GCS activity *in vitro* ^2, 32, 42, 43^, and for tracking Cer translocations in cells^36, 43^. In the current work, it was found that NBD C6-Cer was efficiently taken up, and served as an intracellular substrate along with endogenous ceramides for glycosylation by GCS in cancer cells (Figs 2a and 3a), in dispersed cells of tissues (Figs 4a and 5a), and even in all tissues of live mice simultaneously (Figs 6 and 7). Notably, NBD C6-Cer was well distributed and taken up by tumor and other tissues upon systemic delivery via Cer-RUB nanomicelles (Fig. 6a). NBD C6-Cer quickly accumulated (~15 min) in cells (Fig. 2c), and intracellular amounts correlated linearly with concentrations of NBD C6-Cer added (Fig. 2b). By comparison, NBD C6-Cer accumulation in liver was seen to be delayed after intraperitoneal injection (Fig. 6c). Nonetheless, intra-organ amounts correlated well with doses injected (Fig. 6b).

Secondly, for many enzymes, including GCS, relatively little is known concerning kinetic characteristics under actual *in-vivo* conditions. Our investigations were aimed at ascertaining whether or not NBD Cer incorporation could serve as a viable and valid proxy for endogenous GlcCer production in ways that would enable characterization of enzymatic activity within cells as normally situated and functioning in the tissues of live animals, as opposed to *in-vitro* experiments under well-controlled conditions that generally include purified enzyme, optimal buffered media, and well-defined amounts of substrate(s) and co-enzymes. For Cer glycosylation in cultured cells, we found that the levels of GlcCer produced correlated linearly with NBD C6-Cer concentrations in incubation media (Figs 2b and 6c) at relatively low concentrations (<2 µM), but then asymptotically approached saturation at higher concentrations in cellular or intra-organ glycosylation for which cell numbers or the amount of tissue are fixed. Thus, GlcCer production was in accord with Michaelis-Menten kinetics behavior^44, 45^ (Figs 2b and 6c), similarly to what was seen for enzymatic reactions carried out *in vitro* using GCS prepared from PC12 rat cells^42^. In those laboratory-controlled reactions, GlcCer production increased with incubation time, reaching a plateau after 60 min^42^. In the native environment of cells, GlcCer production was seen to increase with incubation time, reflective of the time-course of cellular accumulation, approaching a plateau after 30 min (Fig. 2c). In the livers of mice, GlcCer production similarly increased with time, but reached significantly levels only after an initial lag phase of about 80 min, thereafter increasing strongly and peaking at about 180 min after intraperitoneal injections (Fig. 6d). More interestingly, the values we calculated for K_m_ and V_max_ in native environments were comparable between human GCS in NCI/ADR-RES cancer cells in culture (2.3 µM and 343 fmol/µg-2 h incubations) (Fig. 2b) and mouse GCS in liver following systemic administration (1.8 µM and 148 fmol/µg-3 h incubation). In that regard, we note the 98% identity of the human GCS sequence with that of mouse GCS^46^. Our calculated K_m_ values for human GCS (2.3 µM) and mouse GCS (1.8 µM) under *in-vivo* conditions are relatively lower than those obtained *in vitro* for human GCS derived from transfected rat PC12 cells (7.9 µM)^42^. It has been reported that several other factors, including Cer localization, and the availability of endogenous UDP-glucose and co-enzymes, can affect cellular GCS activities^1^. Although further studies will be needed to fully understand the mechanistic basis for our observed somewhat lower K_m_ values obtained from the *in-vivo* glycosylation assessments (and to completely rule out any unidentified artifacts) vs. ones obtained under fully laboratory-controlled conditions, the present work at least indicates that NBD C6-Cer incorporation provides a reliable and reproducible approach for assessing ceramide glycosylation catalyzed by GCS in cells and in living animals.

In addition to providing a means of directly assessing functional roles played by GCS in cells situated in their actual dynamically changing *in-vivo* environment, a key advantage of a substrate incorporation-based enzymatic assay is its relative simplicity and efficiency as compared with traditional laboratory-controlled ones. With our NBD C6-Cer incorporation-based methodology, we successfully assessed alterations of GCS activity in cultured cancer cells and in cell suspensions prepared from tumors and other tissues (bone marrow, brain, kidney, small intestine, whole blood cells). Intriguingly, with intraperitoneal injections of NBD C6-Cer-RUB nanomicelles in medium, we were able to assess the ceramide glycosylation happening simultaneously in all nine organs tested, including tumor, lung and liver (Fig. 7b; data for serum, brain, bone marrow, kidney, small intestine and colon not shown).

In summary, we devised, developed and validated Cer incorporation-based methodology with HPLC-based detection and quantitation, for assessing *in-vivo* GCS activity. This direct, specific and effective approach can be applied to measure intracellular and intra-organ GCS activities in live cells and tissues, enabling further assessments of the roles played by GCS in diseases. Notably and in particular, our work to date in applying this methodology suggests it may have considerable value in predicting and monitoring therapeutic efficacy of various anticancer drugs, and of other therapies used in the treatment of GCS-related diseases.

## Materials and Methods

### Materials

*N*-[6-[(7-nitro-2,1,3-benzoxadiazol-4-yl) amino]hexanoyl]-d-*erythro*-sphingosine (NBD C6-ceramide), as well as NBD C6-Cer complexed to bovine serum albumin (BSA) (1:4, w/w), were purchased from Invitrogen (Carlsbad, CA). *N*-Hexanoyl-NBD-glucosylsphingosine (NBD C6-GlcCer), *N*-hexanoyl-NBD-galactosylsphingosine (NBD C6-GalCer), *N*-hexanoyl-NBD-lactosylsphingosine (NBD C6-LacCer), and *d-threo*-1-phenyl-2-decanoylamino-3-morpholino-1-propanol HCl (d-PDMP) were purchased from Matreya (State College, PA). A mixed-backbone oligonucleotide against human GCS (MBO-asGCS; 20-mer) and its scrambled control (MBO-SC) were synthesized and purified by, and obtained from, Integrated DNA Technologies (Coralville, IA)^28, 39^. Small interference RNA (siRNA) silencing human globotriaosylceramide synthase (Gb3S) and the scrambled control were purchased from Santa Cruz Biotechnology (Dallas, TX). Anti-human GCS rabbit serum (GCS 6.2) was kindly provided by Dr. D. L. Marks and Dr. R. E. Pagano (Mayo Clinic and Foundation, Rochester, MN)^47, 48^. Rabbit anti-globotriaosylceramide synthase (Gb3S) polyclonal antibody (sc-32950, H-160) was purchased from Santa Cruz Biotechnology^28^. Other chemicals, except as otherwise indicated, were procured from Sigma-Aldrich (St. Louis, MO) or Fisher Scientific (Pittsburgh, PA).

## Cell culture

Drug-resistant NCI/ADR-RES ovary cancer cells^49^ were kindly provided by Dr. Kenneth Cowan (UNMC Eppley Cancer Center, Omaha, NE) and Dr. Merrill Goldsmith (National Cancer Institute, Bethesda, MD). Cells were maintained in RPMI-1640 medium (Invitrogen) supplemented with 10% fetal bovine serum (FBS), 100 units/mL penicillin, 100 µg/ml streptomycin, and 584 mg/liter l-glutamine. Drug-sensitive A2780 ovarian cancer cells were purchased from ATCC (Manassas, VA)^26^ and were cultured in RPMI-1640 medium containing 10% FBS, 100 units/ml penicillin, 100 µg/ml streptomycin, and 584 mg/liter l-glutamine. Human colon cancer SW48/TP53 (R273H/^+^) cells were purchased from Horizon Discovery (HD 103–008, Waterbeach, Cambridge, UK)^41, 50^. Cells of the OVCAR-3 ovarian cancer line were purchased from ATCC (Manassas, VA). SW48 and OVCAR-3 cells were cultured in RPMI-1640 medium containing 10% FBS, 100 units/mL penicillin, 100 mg/mL streptomycin, and 584 mg/liter l-glutamine, while SW48/TP53 cells were cultured in 10% FBS RPMI 1640 medium including 2 mM l-glutamine, 25 mM sodium bicarbonate, 800 µg/ml geneticin (G418), 100 units/mL penicillin and 100 mg/mL streptomycin. Cells were maintained in an incubator humidified with 95% air and 5% CO_2_ at 37 °C. The cell lines were analyzed by short tandem repeat (STR) profiling (Gene Resources Core Facility, John Hopkins University) and compared against publically available databases (DSMZ and ATCC).

### Cellular ceramide glycosylation and lipid extraction

Cellular Cer glycosylation and lipid extraction were performed as described previously, with modification^28, 37^. Briefly, cells (1 × 10^6^ cells/100-mm petri dish) were grown in 10% FBS RPMI-1640 medium for 24 h. To inhibit GCS enzyme activity, NCI/ADR-RES cells were cultured in 5% FBS RPMI-1640 medium containing 10 µM of d-PDMP for an additional 48 h, as described previously^37^. Cells were exposed to 100 nM of MBO-asGCS in 5% RPMI-1640 medium after transfection with Lipofectamine 2000 for an additional 48 h^28, 39^. For assessing cellular Cer glycosylation, the treated NCI/ADR-RES cells (10^6^ cells/reaction) or A2780 cells were harvested with scrapers and incubated with 2.0 µM of NBD C6-Cer (complexed to BSA) in 1% BSA RPMI-1640 medium (200 µl) at 37 °C for 2 h, with gentle shaking (40 rpm). After rinsing with ice-cold PBS (pH 7.4, three times), cells suspended in ice-cold acidic methanol (acetic acid: methanol, 1:50, v/v) were transferred into glass vials (1.3 ml), and mixed with chloroform (1.3 ml) and water (1.3 ml) to extract cellular lipids. After separation, the organic lower phase was collected and evaporated to dryness, and the residue was then stored at −20 °C until further analysis.

Cellular NBD-sphingolipid accumulation during glycosylation was also viewed with fluorescence microscopy. After incubation with NBD-Cer under the above-described conditions, cells (~100,000 cells/100 µl) were spread onto slides to make smear slides. After fixation with heating and methanol, rehydrated cells were mounted with DAPI (4′,6-diamidino-2-phenylindole) and observed (200× magnification) using an EVOS FL cell imaging system with color CCD camera (Life Technologies, Grand Island, NY). Full length representative images are listed in the Supplementary Information.

### HPLC assay for GCS activity

The HPLC separation and detection of NBD sphingolipids was accomplished as described previously, with modification^32, 51^. Briefly, extracted lipids from cells and tissues were dissolved in 100 µl of chloroform/methanol/*ortho*-phosphoric acid (80:20:0.1, v/v/v), in a manner such that each sample contained approximately equal fluorescence (200 FU in 100 µl). A 5-µl aliquot of each sample was loaded by autosampler injection onto a normal-phase silica column (5 µm ZORBAX Rx-SIL, 4.6 mm × 250 mm). The HPLC system (1220 Infinity LC Gradient System VL) was equipped with an Agilent 1260 fluorescence detector (Agilent, Santa Clara, CA). NBD sphingolipids were eluted by linear gradient (0–14 min, 1 ml/min) formed with solvent system A (chloroform/methanol/*ortho*-phosphoric acid) (80:20:0.1, v/v/v) and solvent system B (chloroform/methanol/H_2_O/*ortho*-phosphoric acid) (60:34:6:0.1, v/v/v/v). NBD fluorescence was detected using excitation and emission wavelengths of 470 and 530 nm, respectively. The fluorescent peaks for NBD sphingolipids were identified by comparing their retention times with those of standards. Individual NBD C6-sphingolipids or an equimolar mixture (1:1:1, fmol/fmol/fmol) of NBD C6-Cer, C6-GlcCer and C6-LacCer served as standards, unless otherwise indicated. The amounts of GlcCer and Cer were quantitated against respective standard curves. Constants of linear correlation, cellular accumulation, and enzyme kinetics constants (K_m_, V_max_) were calculated using the Prism v5 program (GraphPad software, San Diego, CA). Each sample was analyzed at least three times, and reported sphingolipid levels were as normalized against total cellular proteins.

### ESI/MS/MS analysis of sphingolipids

Endogenous sphingomyelin molecular speciation was accomplished with a Surveyor/TSQ Quantum LC/MS system with triple-quadrupole mass spectrometer, operating in a Multiple Reaction Monitoring (MRM) positive ionization mode, as described previously^52–54^. Total cells, spiked with internal standards, were extracted with ethyl acetate/isopropanol/water (60:30:10, v/v). The extracts were evaporated to dryness and reconstituted in 100 μl of methanol. Samples were injected and gradient-eluted from a BDS Hypersil C8 column (150 × 3.2 mm, 3 μm particle size) with a 1.0 mM methanolic ammonium formate/2 mM aqueous ammonium formate mobile phase system. The peaks for the target analytes and internal standards were collected and processed using the Xcalibur software. Calibration curves were constructed by plotting peak area ratios of synthetic standards, representing each target analyte, to a corresponding closely analogous internal standard. The reported levels of sphingolipids in samples, expressed as fmol/µg protein, are as calculated by linear regression, normalized against total cellular protein.

### Western blotting

Western blotting was carried out as described previously^26, 28, 38^. Briefly, cells (3 × 10^6^ cells/100 mm dish) were harvested after treatments, using NP40 cell lysis buffer (Biosource, Camarillo, CA). Equal amounts of proteins (50 µg/lane) were resolved using 4–20% gradient SDS-PAGE (Invitrogen). The transferred blots were blocked with 5% fat-free milk PBS and immunoblotted with anti-GCS serum or anti-Gb3S antibody at 1:500 dilutions, at 4 °C overnight. The immunoblots were detected by using enzyme-linked chemiluminescence (ECL) with a SuperSignal West Femto substrate (Thermo Fisher Scientific). Glyceraldehyde-3-phosphate dehydrogenase (GAPDH) served as a loading control. Relative protein levels are presented as optical densities normalized against those of GAPDH from three blots. Full length representative blots are listed in the Supplementary Information. The amounts of proteins were quantitated using the bicinchoninic acid protein assay (Pierce, Rockford, IL), with BSA as a standard.

### Preparation of NBD Cer-RUB nanomicelles

To enhance water solubility of NBD C6-Cer for Cer glycosylation studies in mice, NBD C6-Cer (Avanti Polar Lipids, Alabaster, AL) was complexed with rubusoside (RUB) (1:100, w/w) to generate NBD-Cer-RUB micelles, as described previously^55^. Briefly, NBD C6-Cer was dissolved in an ethanol solution containing RUB (140 g:100 ml), and this solution was then passed through a 0.45-µm nylon filter. The resulting clarified NBD Cer-RUB solution was evaporated and lyophilized to generate an NBD C6-Cer-RUB complex in powder form. The NBD Cer-RUB complex powder, which was shown to be stable at room temperature for at least four weeks, was stored at 4 °C, protected from light, for longer-term availability. Prior to use, the powder was equilibrated to room temperature, and then reconstituted into aqueous solution.

The morphology of NBD C6-Cer-RUB was characterized by transmission electron microscopy (TEM, JEOL JEM-1011) with an accelerating voltage of 80 kV. Samples for TEM were prepared by adding a drop of the solution containing NBD Cer-RUB complex (3–10%) onto a TEM grid, and allowing the solvent to evaporate. Ultraviolet and visible (UV/vis) spectra were recorded in a 1-cm-width cuvette at room temperature using a GenesysTM 10 spectrometer. The average particle size is 30 ± 5 nm, measured and calculated from all particles in three different TEM images.

### Tumor treatments and Cer glycosylation in mice

All animal experiments were approved by the Animal Care and Use Committee of the University of Louisiana at Monroe (ULM) (Protocol# 16DEC-YYL 01) and were conducted in strict accordance with good animal practice as defined by NIH guidelines. Tumor-bearing mice were studied as described previously^28, 39^. Briefly, athymic nude mice (*Foxn1* ^*nu*^ */Foxn1* ^+^, 4–5 weeks, female) were purchased from Harlan (Indianapolis, IN) and maintained in the vivarium at ULM. Cell suspensions of SW48/TP53 or OVCAR-3 (3–5 passages, 1 × 10^6^ cells in 20 µl medium per mouse) were inoculated subcutaneously into the left flanks of mice. Treatments with d-PDMP (4.0 mg/kg, intraperitoneal, every 3 days) and doxorubicin (200 µg/kg, intraperitoneal, every 6 days) were started once tumors reached ~5 mm in diameter, and continued for 30 days.

Cell suspensions of tumors and other tissues of mice inoculated with SW48/TP53 cells were prepared immediately after their resections as described previously, with modification^37, 38, 56^. Tissues (~1–2 mg) were pieced (<1 mm) and incubated at 37 °C for 1 h in 100 µl of RPMI-1640 medium containing collagenase IV (500 units/ml). Glycosylation was carried out in 200 µl of a cell suspension (1 × 10^6^ cells) in 1% BSA RPMI-1640 medium containing NBD C6-Cer (2 µM), at 37 °C for 2 h with gentle shaking (30 rpm). Cellular NBD sphingolipids were observed on smear slides by fluorescent microscopy during incubation. Lipids from these samples were extracted and analyzed as described above.

Once tumor sizes in mice inoculated with OVCAR-3 cells reached ~5 mm, NBD Cer-RUB (1 mg/kg in RPMI-1640 medium) was intraperitoneally injected. Three hours after administration, tumors and other tissues (~1 mg) were collected, pieced, and sonicated in 200 µl of RPMI-1640 medium. Tissue lipids were extracted and further analyzed, as described above.

### Data analysis

All experiments were repeated 2 or 3 times. Data are expressed as mean ± SD. Two-tailed Student’s *t* tests and ANOVA tests were used to compare the continuous variables in groups, using the Prism v7 program (GraphPad Software, La Jolla, CA). All *p* < 0.05 comparisons were regarded as statistically significant.

### Data availability

All data generated or analyzed during this study are included in this published article and its Supplementary Information file.

## Electronic supplementary material

Supplymentary Information
