# Supplementary material for: Incorporation of Fluorescence Ceramide-Based HPLC Assay for Rapidly and Efficiently Assessing Glucosylceramide Synthase In Vivo
Source: Sci Rep. 2017 Jun 7;7:2976. doi: 10.1038/s41598-017-03320-9 (PMC5462733; doi:10.1038/s41598-017-03320-9)
Supplement: Supplementary file 1 — Supplymentary Information [file 41598_2017_3320_MOESM1_ESM.pdf]

Supplementary Information

Incorporation of Fluorescence Ceramide-Based HPLC Assay for Rapidly and Efficiently Assessing Glucosylceramide Synthase *In Vivo*

Sachin K. Khiste[1], Salman B. Hosain[1], Yixuan Dong[2], Mohammad B. Uddin[1], Kartik R. Roy[1], Ronald A. Hill[1], Zhijun Liu[2], Yong-Yu Liu[1,*]

[1]Department of Basic Pharmaceutical Sciences, University of Louisiana at Monroe, Monroe, Louisiana
[2]School of Renewable Natural Resources, Louisiana State University, Baton Rouge, Louisiana

Supplementary Figures

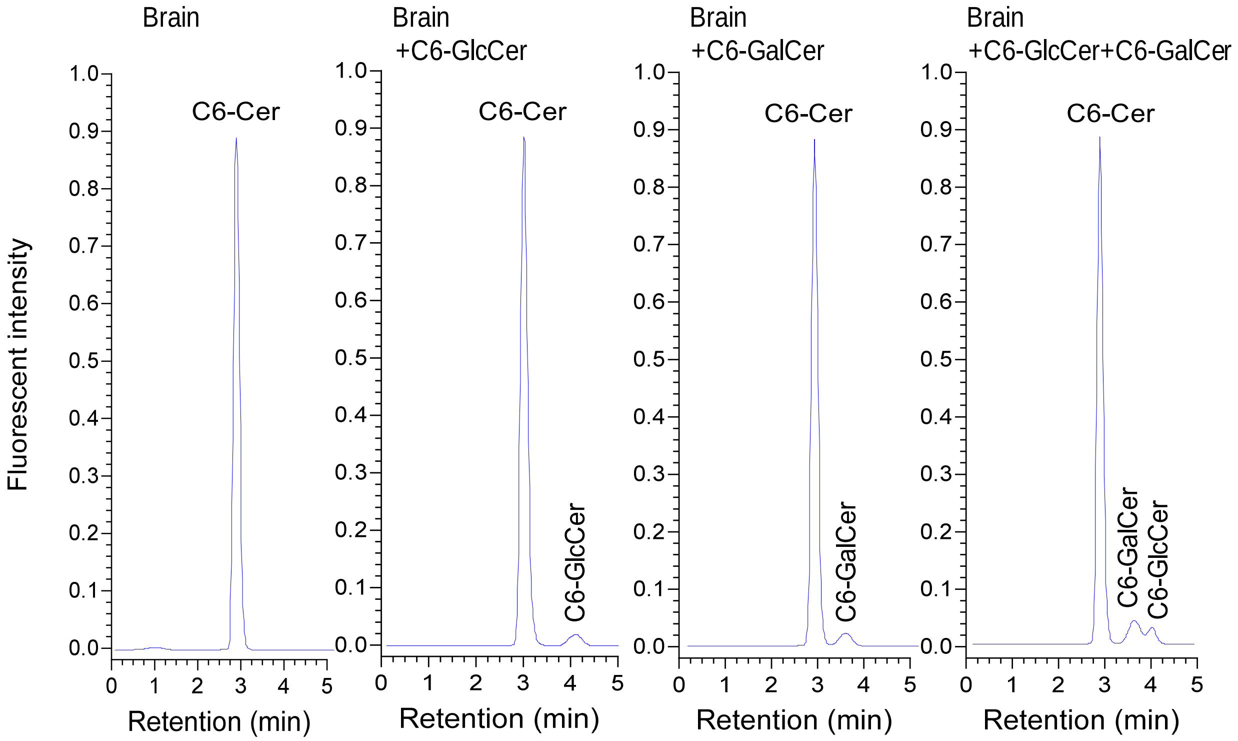

Figure S1. HPLC chromatograph of brain samples with NBD C6-glucosylceramide (C6-GlcCer) and NBD C6-galactosylceramide (C6-GalCer). Lipids were extracted from mouse brain after 3 hr NBD C6-ceramide administration (1 mg/kg, i.p) and analyzed with HPLC. C6-GalCer (125 nmol/5 μl) and C6-GlcCer (125 nmol/5 μl) added to brain samples were effectively separated and appeared at 3.6 and 4.1 min, respectively.

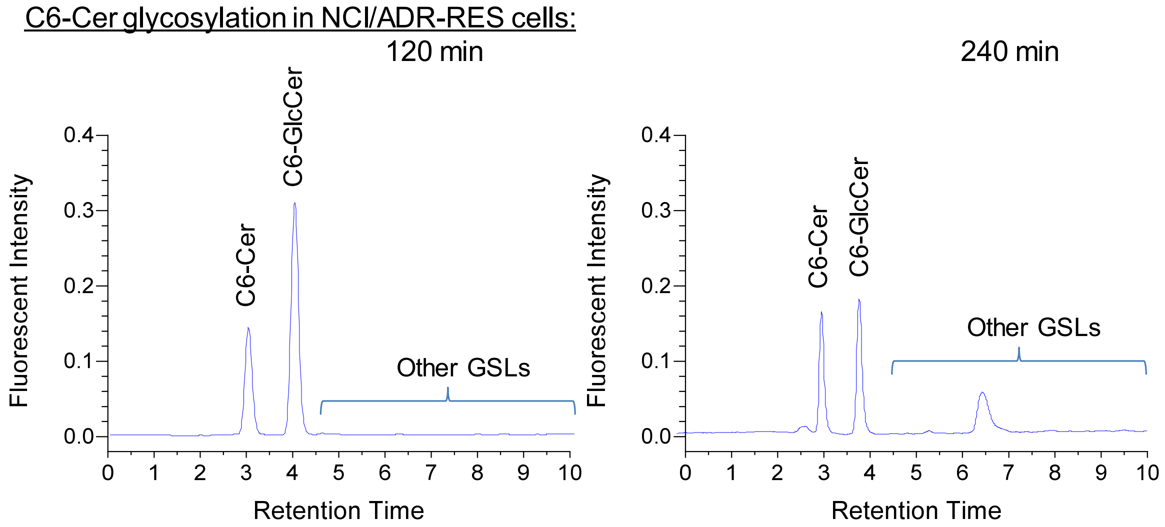

Figure S2. Ceramide glycosylation in NCI/ADR-RES cells after 2 and 4 hours of incubation with NBD C6-ceramide (NBD C6-Cer, 2.0 μM). Cellular levels of NBD C6-glucosylceramide (C6-GlcCer) were decreased after 4 h incubation, compared to these after 2 h; however, the levels of other NBD C6-glycosphingolipids (GSLs) that have never been characterized in this study increased.

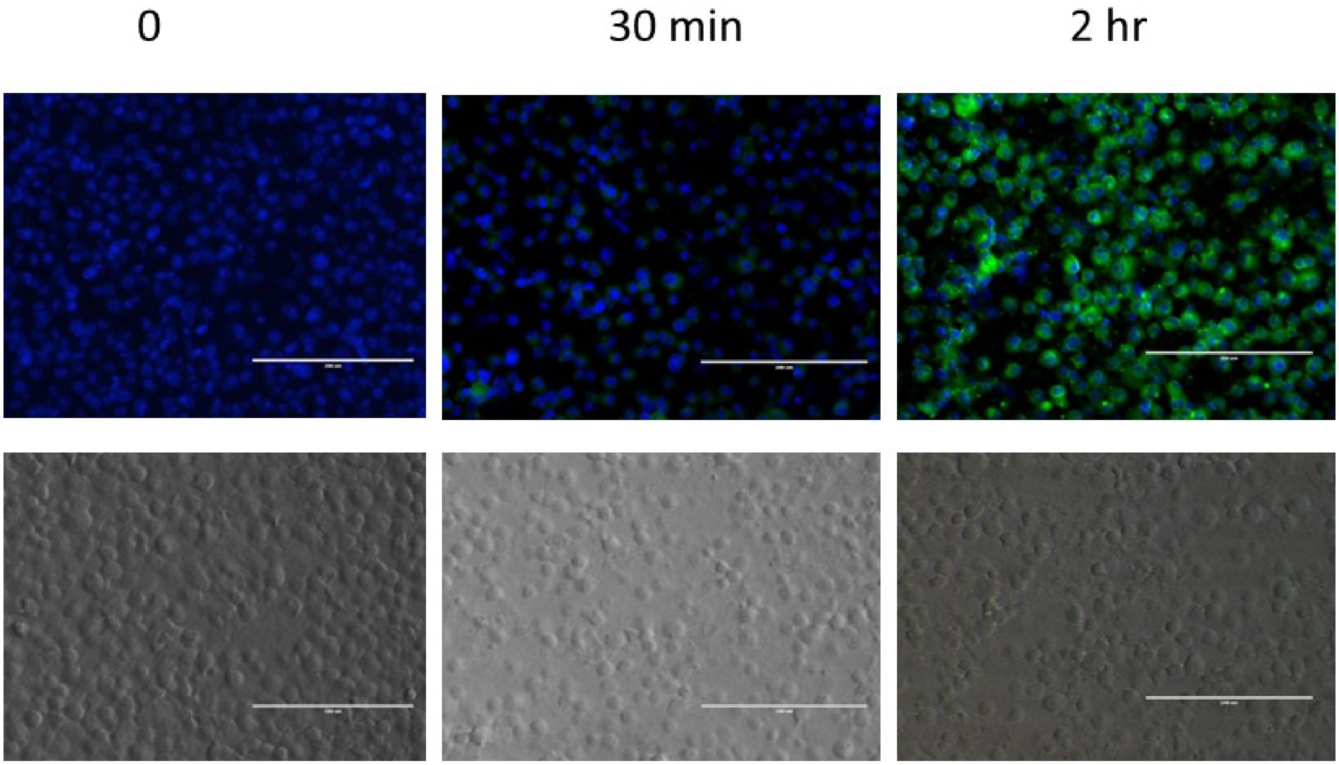

Figure S2a. Full length microscopic images of fluorescent NBD-sphingolipids in cells after incubations of 2 μM NBD C6-Cer at different time point. Green, NBD-sphingolipids; Blue, DAPI-nuclei. Images (200x magnification) were captured using an EVOS FL cell imaging system.

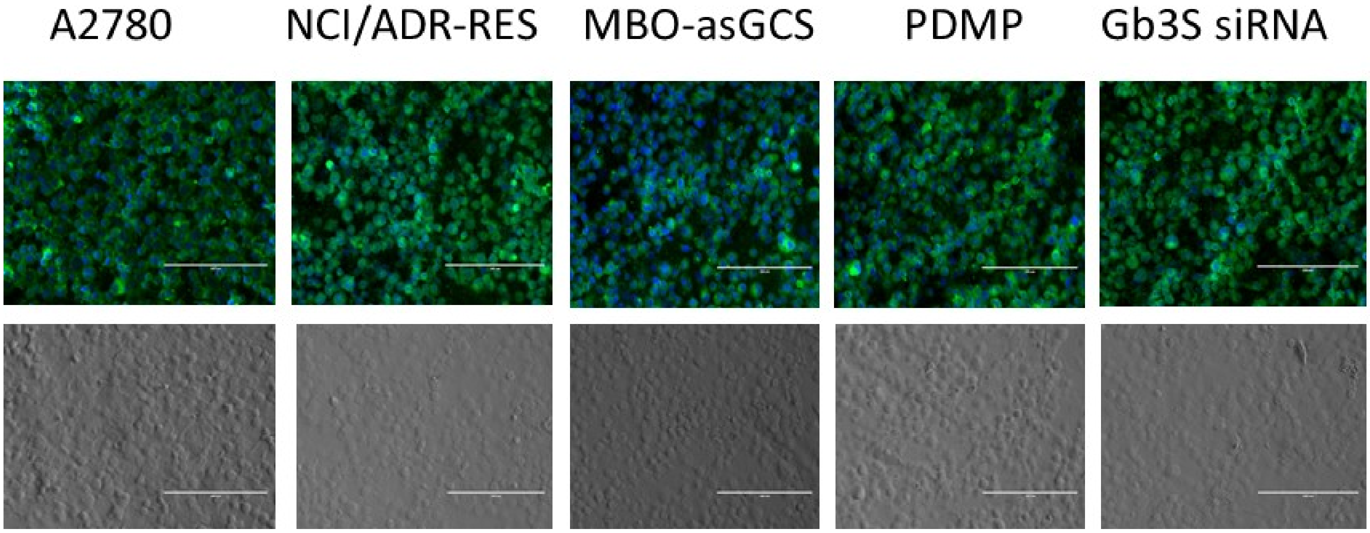

Figure S3a. Full length microscopic images of fluorescent sphingolipids in cells after incubation with NBD C6-Cer. Green, NBD-sphingolipids; Blue, DAPI-nuclei. Images (200x magnification) were captured using an EVOS FL cell imaging system.

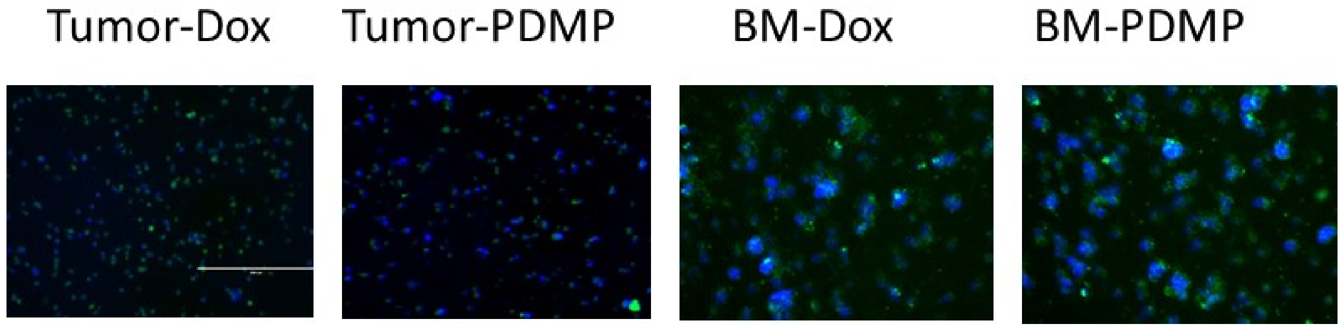

Figure S4a. Full length microscopic images of fluorescent sphingolipids in Mice bearing SW48/TP53 tumors and bone marrow were treated with doxorubicin (Dox) alone or combined with PDMP cells after incubation with NBD C6-Cer. Green, NBD-sphingolipids; Blue, DAPI-nuclei. Images (200x magnification) were captured using an EVOS FL cell imaging system

Supplementary Information: Western Blots

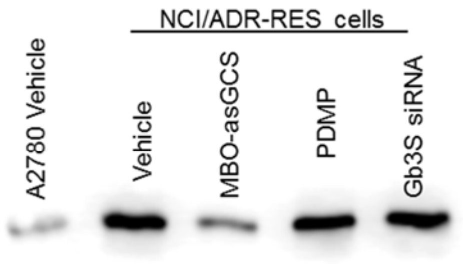

Figure S3d. Full length Western blot image of cell GCS. Equal amounts of proteins (50 µg protein/lane) extracted were resolved and then immunoblotted with GCS antibody.

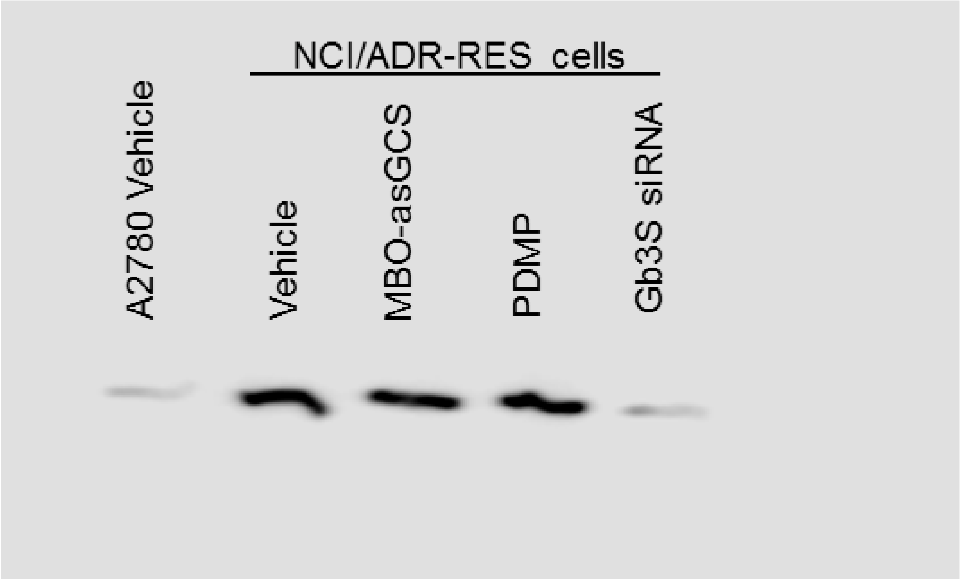

Figure S3d. Full Western blot image of cell Gb3S. Equal amounts of proteins (50 µg protein/lane) extracted were resolved and then immunoblotted with Gb3S antibody.

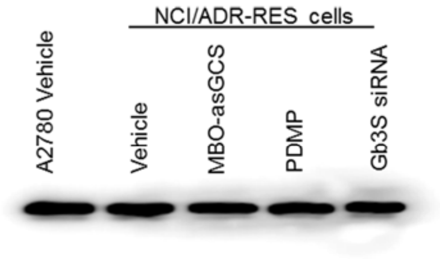

Figure S3d. Full Western blot image of cell GAPDH. Equal amounts of proteins (50 μg protein/lane) extracted were resolved and then immunoblotted with GAPDH antibody.

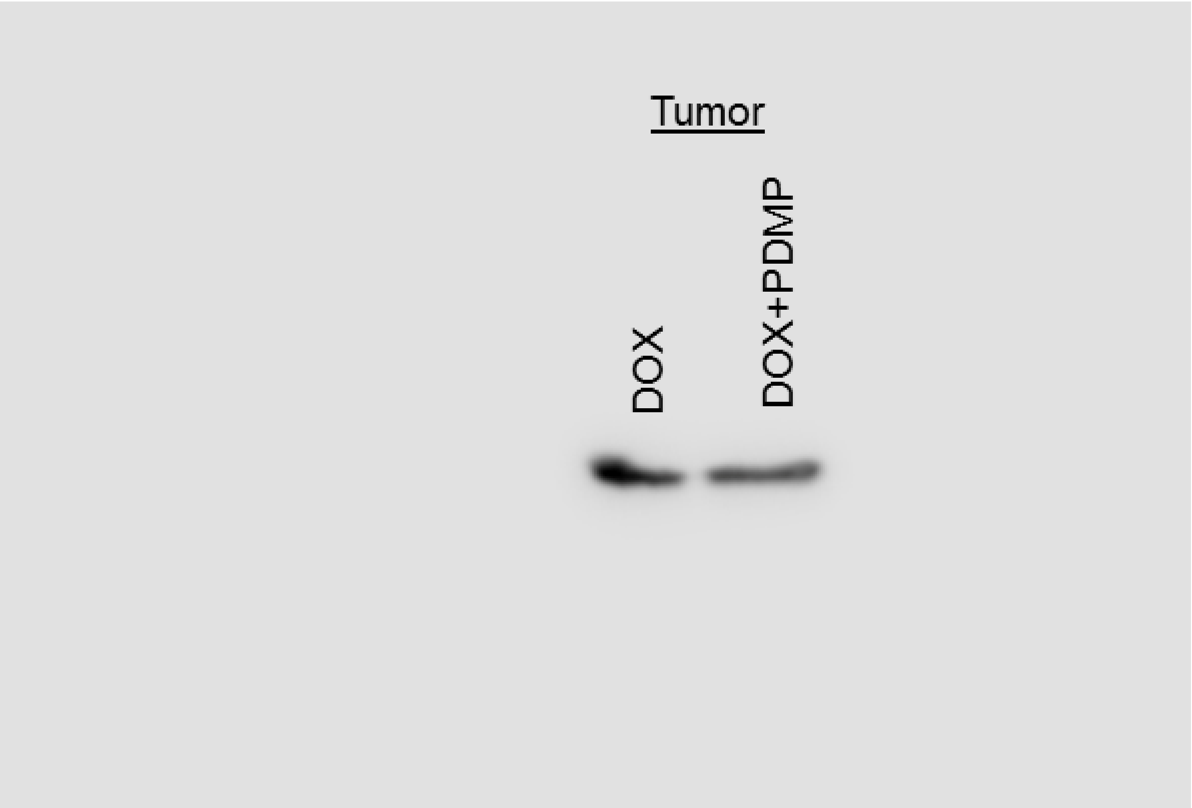

Figure S4c. Full Western blot image of tumor tissue GCS. Equal amounts of proteins (50 μg protein/lane) extracted were resolved and then immunoblotted with GCS antibody.

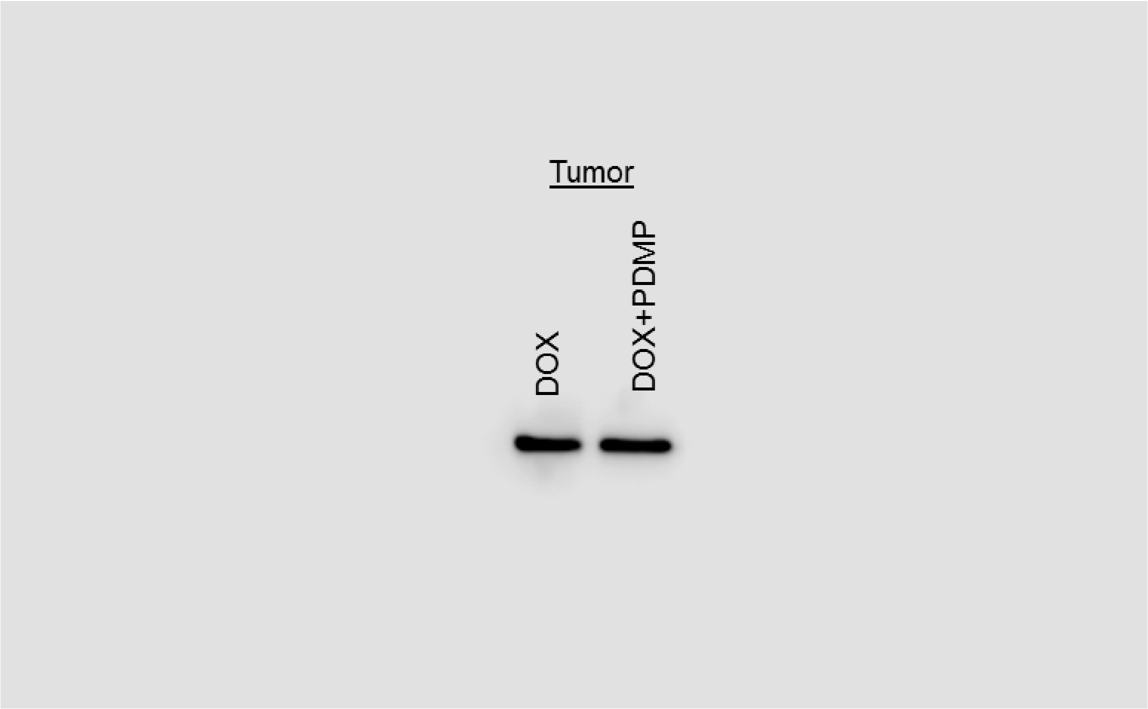

Figure S4c. Full Western blot image of tumor tissue GAPDH. Equal amounts of proteins (50 µg protein/lane) extracted were resolved and then immunoblotted with antibodies for GAPDH.

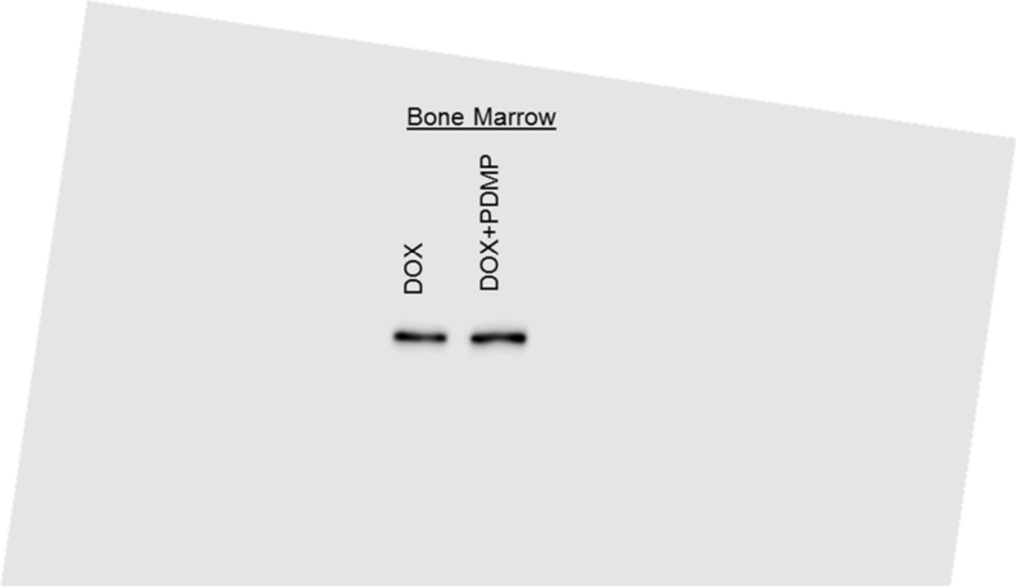

Figure S4c. Full Western blot image of Bone Marrow GCS. Equal amounts of proteins (50 µg protein/lane) extracted were resolved and then immunoblotted with GCS antibody.

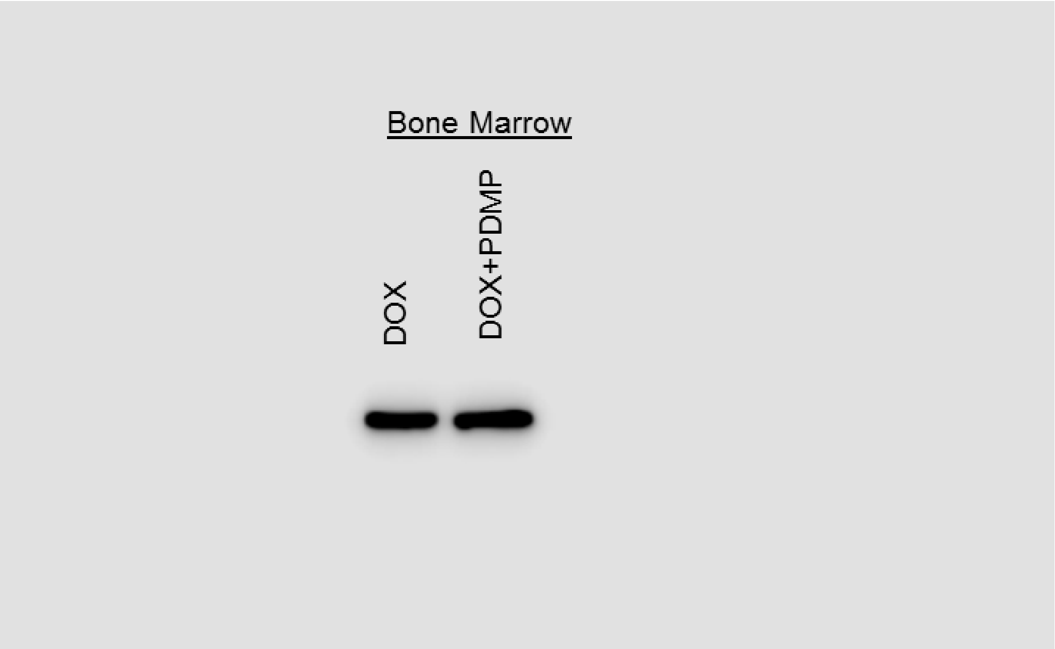

Figure S4c. Full Western blot image of Bone Marrow GAPDH. Equal amounts of proteins (50 µg protein/lane) extracted were resolved and then immunoblotted with GAPDH antibody.

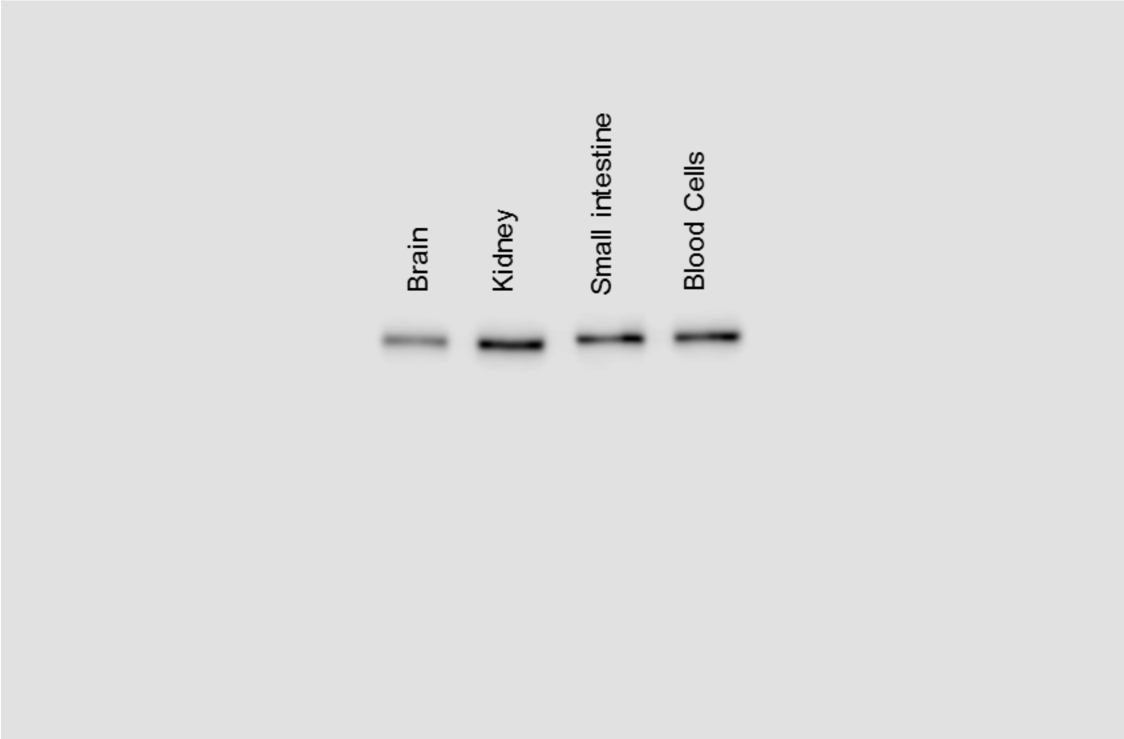

Figure S5c. Full Western blot image of respective mice tissues GCS. Equal amounts of proteins (50 µg protein/lane) extracted were resolved and then immunoblotted with GCS antibody.

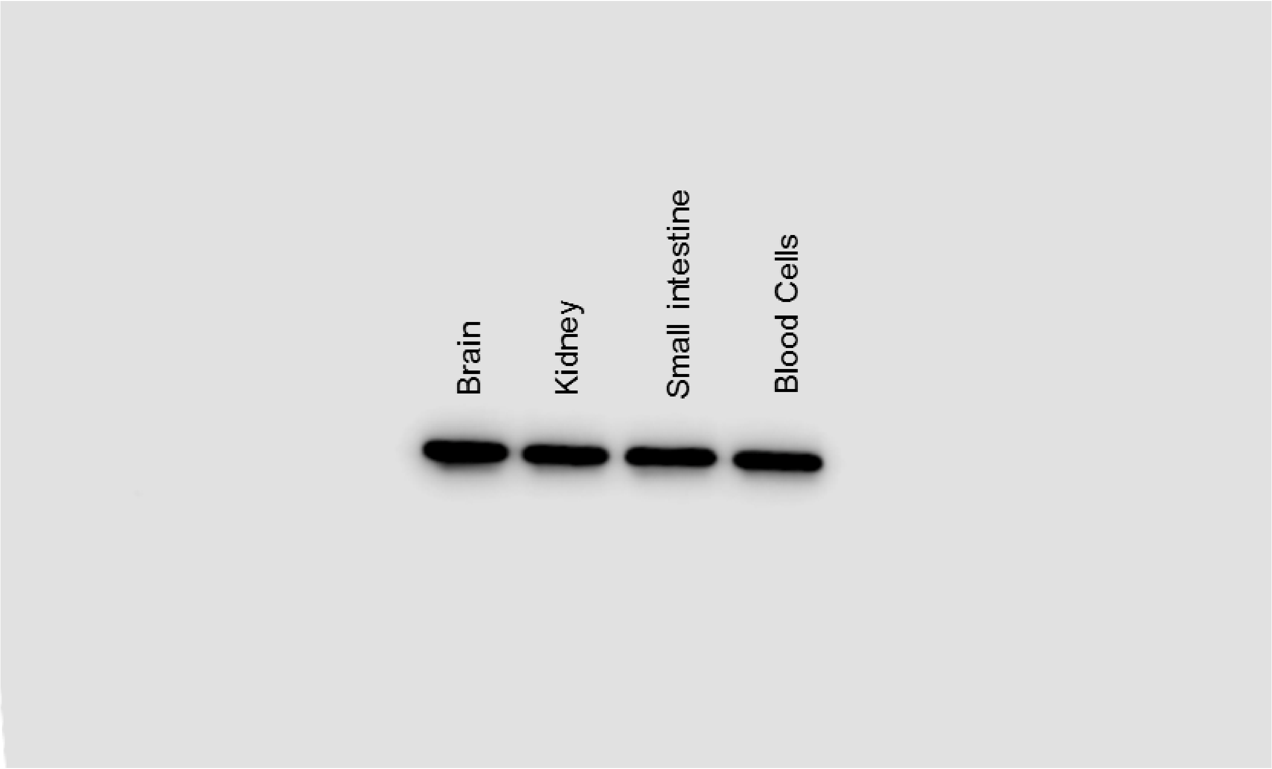

Figure S5c. Full Western blot image of mice tissues GAPDH. Equal amounts of proteins (50 µg protein/lane) extracted were resolved and then immunoblotted with GAPDH antibody.

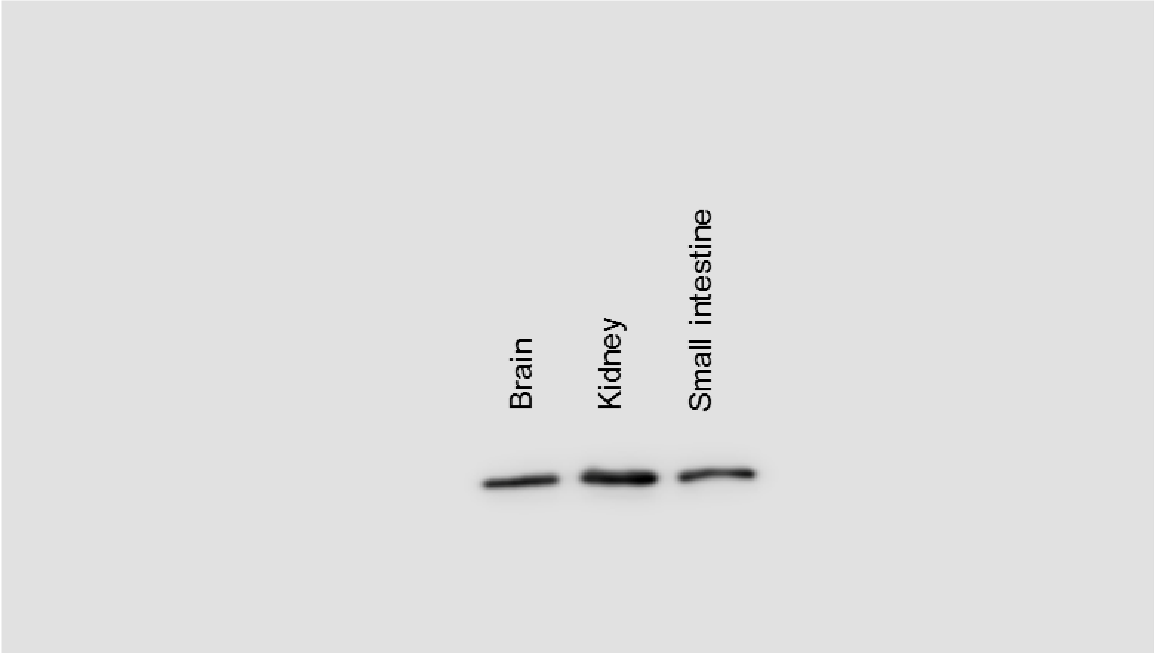

Figure S7c. Full Western blot image of mice tissues GCS. Equal amounts of proteins (50 µg protein/lane) extracted were resolved and then immunoblotted with GCS antibody

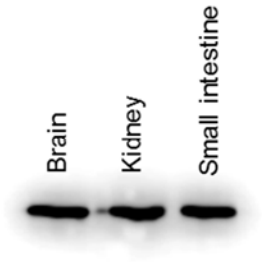

Figure S7c. Full Western blot image of mice tissues GAPDH. Equal amounts of proteins (50 μg protein/lane) extracted were resolved and then immunoblotted with GAPDH antibody.
